# Impact of Bottom Trawling on Deep-Sea Sediment Properties along the Flanks of a Submarine Canyon

**DOI:** 10.1371/journal.pone.0104536

**Published:** 2014-08-11

**Authors:** Jacobo Martín, Pere Puig, Pere Masqué, Albert Palanques, Anabel Sánchez-Gómez

**Affiliations:** 1 Institut de Ciències del Mar, Consejo Superior de Investigaciones Científicas (ICM-CSIC), Barcelona, Spain; 2 Institut de Ciència i Tecnologia Ambientals & Departament de Física, Universitat Autònoma de Barcelona, Bellaterra, Spain; Auckland University of Technology, New Zealand

## Abstract

The offshore displacement of commercial bottom trawling has raised concerns about the impact of this destructive fishing practice on the deep seafloor, which is in general characterized by lower resilience than shallow water regions. This study focuses on the flanks of La Fonera (or Palamós) submarine canyon in the Northwestern Mediterranean, where an intensive bottom trawl fishery has been active during several decades in the 400–800 m depth range. To explore the degree of alteration of surface sediments (0–50 cm depth) caused by this industrial activity, fishing grounds and control (untrawled) sites were sampled along the canyon flanks with an interface multicorer. Sediment cores were analyzed to obtain vertical profiles of sediment grain-size, dry bulk density, organic carbon content and concentration of the radionuclide ^210^Pb. At control sites, surface sediments presented sedimentological characteristics typical of slope depositional systems, including a topmost unit of unconsolidated and bioturbated material overlying sediments progressively compacted with depth, with consistently high ^210^Pb inventories and exponential decaying profiles of ^210^Pb concentrations. Sediment accumulation rates at these untrawled sites ranged from 0.3 to 1.0 cm y^−1^. Sediment properties at most trawled sites departed from control sites and the sampled cores were characterized by denser sediments with lower ^210^Pb surface concentrations and inventories that indicate widespread erosion of recent sediments caused by trawling gears. Other alterations of the physical sediment properties, including thorough mixing or grain-size sorting, as well as organic carbon impoverishment, were also visible at trawled sites. This work contributes to the growing realization of the capacity of bottom trawling to alter the physical properties of surface sediments and affect the seafloor integrity over large spatial scales of the deep-sea.

## Introduction

Bottom trawling is a fishing technique that consists in pulling nets in contact with the seafloor to capture bottom-dwelling animals of commercial interest. To make the fishing gear operative, heavy devices such as lead rolls, bobbins, sweeplines, tickler chains or otter boards are used which cause widespread mortality of benthos, stirring and resuspension of sediments, changes in oxygen penetration depths and sediment geochemistry and a general destructuration of benthic habitats [1], [2], [3], [4]. This practice has been extended to new and deeper grounds during the last few decades due to the exhaustion of shallow water fish stocks and taking advantage of technological improvements and subsidies that have encouraged the exploitation of previously inaccessible deep-sea resources [5], [6].

Most previous studies addressing the impacts of trawling on bottom sediments have focused on relatively shallow settings affected by energetic hydrodynamic forcing such as storm waves and tides. The deep (>200 m) seafloor is in general subjected to a limited degree of physical disturbance and sediment remobilization by natural processes. In such a context, the relative weight of anthropogenic disturbances can be overwhelming and long-lasting [7], [8]. Human activities such as deep-sea mining, ocean dredge spoil dumping, laying of undersea cables, shipwrecks or warfare can occasionally lead to acute disturbances on the seafloor, but at present commercial bottom trawling largely leads the list of man-driven impacts on bottom sediments, due to the combination of its recurrence, intensity, mobility and wide geographical extent [9], [10].

**Figure 1:**
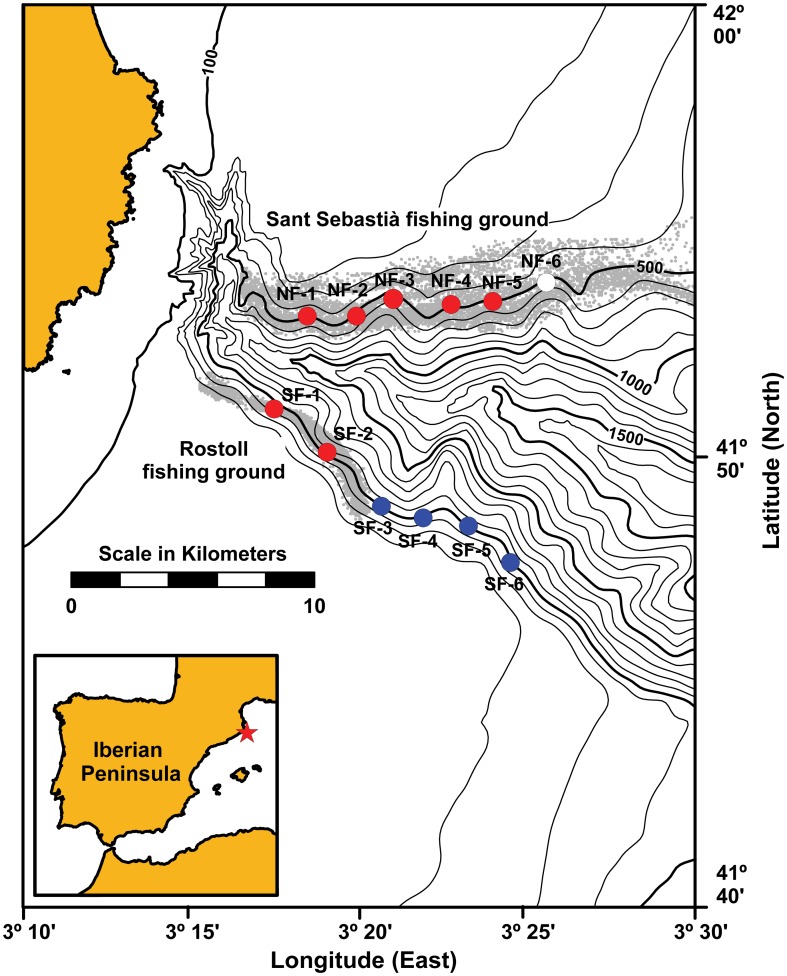
Bathymetric chart of La Fonera (or Palamós) Canyon in the NW Mediterranean Sea. The main fishing grounds active on the canyon flanks are marked with grey points corresponding to Vessel Monitoring System (VMS) positions from Palamós harbour trawlers for the period 2007–2010 (see Puig et al., 2012 for details). Coring stations visited in this study are labelled based on their location in the northern flank (NF) or southern flank (SF), increasing their numbering with distance from shore. For further clarity, blue dots have been assigned to untrawled (control) and red dots to trawled sampling sites. Site NF-6 could not be sampled due to the extreme coarseness and compaction of surface sediments (see the text for details).

La Fonera (or Palamós) submarine canyon (Fig. 1) is one of the most prominent submarine canyons incising the NE Spanish continental margin. The bifurcated canyon head opens very close to the coast, while its upstream (northern) flank cuts the continental shelf and upper slope transversally to the mean SW regional flow. In this way, the canyon intercepts and channelizes further offshore suspended particles that are transported by along-shore currents, resuspended during storms or episodes of dense shelf water cascading [11], [12], [13].

An otter trawl fishery targeting the deep water shrimp *Aristeus antennatus* operates along the flanks of La Fonera Canyon on a daily basis and year-round at depths of 400–800 m [14]. The fishery has been active since the first half of the 20^th^ century, but the engine power of the fishing boats was negligible until the 1960s. In little more than a decade (roughly the 1970s decennium), total power of the trawling fleet increased from less than 1400 hp to more than 16000 hp [15]. This allowed increases in the size and weight of the trawling gears, as well as the length and duration of fishing hauls, overall resulting in an enhanced capacity to impact the seafloor [16]. At present, all the northern flank of La Fonera Canyon (stations prefixed with NF in Fig. 1) has been extensively trawled, while the southern flank (stations prefixed with SF in Fig. 1) presents a trawled proximal part and a distal one that remains virtually unexploited, which will be hereafter termed the control area.

Previous studies in this submarine canyon have shown that the passage of bottom trawling gears along the canyon flanks can trigger sediment gravity flows [17], which transport sediments from the fishing grounds into the canyon axis [11], [18]. This human-induced chronic alteration of the seafloor has reshaped the contemporary sediment dynamics in the canyon, enhancing sedimentation rates in the lower canyon [15] and smoothening seafloor features on fishing grounds [10]. These studies [10], [11], [15], [17], [18] addressed the effects of trawling disturbance of bottom sediments indirectly, either from observations of the down-canyon propagation of trawling-induced resuspension or regarding its consequences on seafloor morphology at large spatial scales. Recently, [19] addressed the effects of trawling impacts on the biochemical composition of sediments along the canyon flanks and [20] analyzed the impacts on their meiofauna composition. So far, a dedicated study on the effects of trawling on the physical properties and erosion/accumulation patterns of sediments in the fishing grounds, compared to untrawled slope environments at the same depth, had not yet been carried out.

With the aim to study the degree of alteration of surface sediments exposed to recurrent trawling, we sampled La Fonera Canyon flanks with a multicorer, including stations in the fishing grounds as well as control sites unaffected by trawling. Sediment cores were analyzed to obtain vertical profiles of grain-size, dry bulk density and organic carbon content. The occurrence of the natural radionuclide ^210^Pb at each station was also investigated, in order to infer the prevalence of mixing, accumulation or erosion and to estimate sediment accumulation rates where possible.

## Methods

A KC Denmark A/S 6-tube (inner diameter 9.4 cm; length 600 mm) multicorer was deployed at 12 stations on the flanks of La Fonera Canyon during cruises HERMIONE-I (May 2011) and HERMIONE-II (October 2011). No specific permits were required for the described field studies. The sediment sampling locations are not privately-owned or protected in any way and the field study did not involve endangered or protected species. The sampling strategy included trawled and control (untrawled) sites along the 500-m isobath on both canyon flanks (Fig. 1).

The distribution of commercial trawling for the period 2007–2010 is illustrated in Figure 1 using the data of the satellite-based Vessel Monitoring System (VMS, see [10] for details). Furthermore, the inspection of fishermen logbooks (e.g., [17]) and historical fishing charts from pre-VMS times show that the main fishing grounds have remained basically the same for decades, only increasing its maximum depth as technical improvements were introduced. Recent studies in the study area have shown clear statistical differences between the sediments collected at trawled and untrawled sites both in terms of its biology and biochemistry [19], [20] as well as in terms of seabed morphology [10].

Coordinates of sampling locations are given in Table 1. Station NF-6 (Fig. 1) had to be abandoned after several failed deployments while successful core sets were extracted from the other 11 sampling stations. The length of the retrieved sediment tubes ranged from 25 to 50 cm. At each coring site, two sediment tubes were selected that fulfilled these two conditions: the sediment–water interface was undisturbed and the two cores were the most similar within the multicorer set in terms of height and external appearance. A vertical slab of sediment was removed from one of this core tubes for X-radiographic analysis. The second undisturbed tube was sliced on-board at 1 cm intervals and the obtained sections freeze-dried for further analysis. Dry weight was obtained with an analytical balance and corrected for salt content, the latter inferred from water content assuming a pore water salinity of 38. Dry bulk density was calculated as the dry mass divided by the total volume of the sample, the latter estimated from water and sediment contents assuming a density of 1.025 g cm^−3^ for sea water and 2.65 g cm^−3^ as average grain density. For granulometric analysis, ∼1 g of dried sediment sample was treated with 20% H_2_O_2_ for 48 h to remove organic matter and then dispersed by adding sodium hexametaphosphate solution overnight. Grain size analysis of the sample thus pre-treated was conducted by laser diffractometry using a Horiba Partica LA-950V2 particle-size analyzer, which provides a wide dynamic range of measurements (0.01–3000 µm) with a 0.6% accuracy and a 0.1% precision. Sediment samples were previously sieved through a 3 mm mesh and the relative percentage of coarser particles incorporated later into the grain-size distribution. For the determination of organic carbon content (as percentage of total dry weight), aliquots of dried sediment were first treated with HCl to remove inorganic carbon, then the remaining carbon in the sample was analyzed with a LECO TruSpec CN auto-analyzer (25 ppm C precision, with a 0.0001 ppm readability).

**Table 1:** Sampling dates and positions and main ^210^Pb parameters of sediment cores retrieved from the flanks of La Fonera Canyon.

Core	Coordinates	Depth	Sampling	Status	Core length	^210^Pb_xs_ horizon	Surface ^210^Pb_xs_	SML	SAR	SR	Inventory
	°E/°N	m	date		cm	cm	Bq kg^−1^	cm	g cm^−2^ y^−1^	cm y^−1^	Bq m^−2^
NF-1	3.307/41.885	475	12/10/2011	**TR**	25	>25	117±9	10?	-	-	>18000
NF-2	3.332/41.886	591	12/10/2011	**TR**	40	>40	128±9	7-15?	-	-	>40000
NF-3	3.349/41.890	511	13/05/2011	**TR**	30	1	5.3±2.6	-	-	-	80±32
NF-4	3.378/41.889	486	12/10/2011	**TR**	35	18	40±4	18	-	-	6200±150
NF-5	3.402/41.891	484	13/05/2011	**TR**	30	9	67±5	9	-	-	3900±140
SF-1	3.291/41.851	463	12/10/2011	**TR**	28	6	62±6	6	-	-	1300±80
SF-2	3.318/41.834	503	13/05/2011	**TR**	34	4-6	191±9	6	-	-	1800±80
SF-3	3.343/41.815	457	12/10/2011	**U**	40	>43	200±9	16	0.74±0.04	1.0±0.1	>46000
SF-4	3.365/41.810	453	12/10/2011	**U**	50	>50	223±15	17	0.51±0.02	0.66±0.02	>44000
SF-5	3.387/41.807	472	13/05/2011	**U**	45	40	214±12	10	0.26±0.02	0.35±0.02	28700±330
SF-6	3.407/41.794	498	12/10/2011	**U**	50	>50	261±16	10	0.39±0.02	0.50±0.02	>38000

Data in the table includes ^210^Pb horizons (penetration depth of excess ^210^Pb), surface mixed layers (SML), sedimentation rates (SR), sediment accumulation rates (SAR) and excess ^210^Pb inventories. In some cores the depth of ^210^Pb/^226^Ra secular equilibrium was not reached and consequently the inventory is a lower estimate. The **TR**awled or **U**ntrawled status of sampling sites is also indicated.

Concentrations of the natural radionuclide ^210^Pb were determined in dried ground samples by counting the alpha-emission of its radioactive product ^210^Po following [21]. ^210^Pb, a decay product of the ^238^U natural radioactive series, is continuously introduced into the marine environment from the atmosphere, through radioactive decay from ^222^Rn emanated from the crust, and is also produced in the water column by decay of dissolved ^226^Ra. Due to its strong affinity with particulate matter, ^210^Pb is removed from the water column by settling particles and tends to accumulate in the sea floor at activities above levels supported by in situ decay of ^226^Ra present in the sediments. The half-life of ^210^Pb (22.3 years) makes it suitable to study accumulation rates of sediments deposited within the past 100–150 years [22], [23]. Excess ^210^Pb concentrations were computed as the difference between measured total ^210^Pb concentrations and the concentration of ^210^Pb in secular equilibrium with its parent radionuclide ^226^Ra (supported ^210^Pb), the latter taken as the concentration of ^210^Pb in the deepest part of the core where the ^210^Pb profile becomes invariant with depth. For some cores, the equilibrium depth was not reached, and we used a mean regional ^210^Pb background of 30±2 Bq kg^−1^, inferred from this and previous studies in the NW Mediterranean [15], [24], [25].

Surface mixed layers (SML) where interpreted from the simultaneous observation of excess ^210^Pb slopes and other indications of the prevalence or absence of mixing (RX opacity, density and grain-size vertical profiles).

A Principal Component Analysis (PCA) from the results obtained in the upper 10 cm of the sediment cores was conducted using IDL (Interactive Data Language) programming language. Four independent variables were used (sand percentage, dry bulk density, organic carbon content and excess ^210^Pb concentration), which were normalized according to their standard deviation before conducting the PCA. Values from missing ^210^Pb measurements in specific sections were obtained from interpolation between contiguous values to fill the data matrix.

## Results

**Figure 2:**
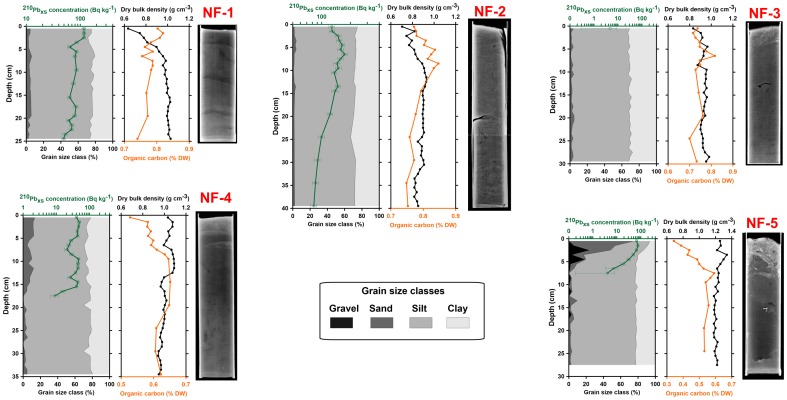
Results from sediment cores collected at the northern flank of La Fonera Canyon. These include, for each sediment core and from left to right: Grain-size distribution by main size classes (grey scale areas); vertical profiles of dry bulk density (purple lines and dots), ^210^Pb excess concentration (green) and negative X-radiographs. When the X-ray images shown are composed of two radiographs, the overlapping point is marked with dotted line. Station names are colored in red and blue for trawled and control sites, respectively (see Fig. 1 for locations).

**Figure 3:**
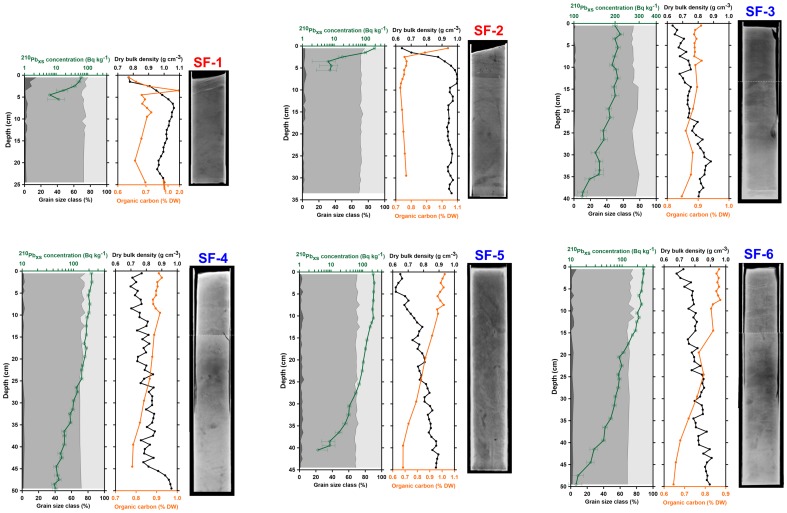
Results from sediment cores collected at the southern flank of La Fonera Canyon. Same as Figure 2.

The vertical profiles of sediment grain-size, dry bulk density, organic carbon content and excess ^210^Pb concentration for each sediment core, together with corresponding X-radiographs are shown in Figures 2 and 3 according to the location of coring sites on the north or south canyon flanks, respectively. Mean ^210^Pb parameters for each sediment core, including excess ^210^Pb horizons, inventories and, when applicable, sediment accumulation rates, are shown in Table 1. The complete dataset used in this work is available as supporting material.

### Density and physical structures in surface sediments

The X-radiographs of sediment cores in general showed poorly structured, bioturbated or mechanically mixed sediments, lacking clear physical structures with the exception of NF-1 where horizontal laminations were visible (Figs. 2, 3).

Dry bulk density oscillated from values close to 0.60 g cm^−3^ at the sediment–water interface of cores NF-1, SF-2 and SF-3 to maxima up to 1.35 g cm^−3^ in core NF-5. At most sampling stations, dry bulk density increased with depth and tended to stabilize around values ∼0.8–1.0 g cm^−3^ towards the bottom of the cores (Figs. 2, 3). Cores from untrawled sites (SF-3 to SF-6) showed a fairly continuous transition from liquefied to denser mud (i.e. from ∼0.6 g cm^−3^ to ∼0.9 g cm^−3^), while at the more land-proximal trawled sites NF-1, NF-2, SF-1, SF-2, this transition was more abrupt with a limit between 5 and 10 cm depth, depending on the particular core, separating two sedimentary units of contrasting densities. These units were also discriminable by visual inspection (see as an example the photograph of the sediment tube of core NF-1 just after collection, Figure S1) and by their different opacity in the X-radiographs. However, this was not the case at more offshore trawled stations NF-3, NF-4 and NF-5, where relatively high densities were present at the sediment–water interface or just a few centimeters below (Figs. 2, 3).

### Grain Size

Surface sediments were for the most part composed of clayey silt with sand as a minor component (<4%), except for the upper sediment unit of stations SF-1, NF-4 and NF-5, where mean sand contents in the 0–10 cm interval were 4.4, 9.9, 17.8%, reaching maximum values (in all cases at the topmost cm) of 14.3, 13.5 and 75.5%, respectively.

The presence of coarser material in the topmost sections of core NF-5 is also notable in the X-radiograph as an upper brighter layer, which also comprised gravel-sized particles composed mainly by fragments of bivalve shells (Fig. 2). Vertical differences in sediment texture were less conspicuous in the rest of the sediment cores. Nonetheless, along the south canyon flank, sand content in the topmost 10 cm of the sediment column was slightly higher in the 2 inshore (trawled) stations SF-1 and SF-2 (4.5 and 2.9%, respectively) than in the 4 offshore (untrawled) stations SF-3, SF-4, SF-5 and SF-6 (0.8, 1.8, 2.3 and 2.2%, respectively).

### ^210^Pb

Excess ^210^Pb concentrations in the sediment–water interface ranged from 5.3±2.6 Bq kg^−1^ at NF-3 to 261±16 Bq kg^−1^ at SF-6 (Table 1). Excess ^210^Pb profiles were observable at all sediment cores with the exception of NF-3, which lacked any unsupported ^210^Pb below the topmost 1 cm (Figs. 2, 3). At stations SF-1, SF-2 and NF-5, excess ^210^Pb was restricted to the upper 5 cm. The horizon of supported ^210^Pb was not reached in cores NF-1, NF-2, SF-3, SF-4 and SF-6, and in these cases the ^210^Pb inventories shown in Table 1 must be regarded as lower estimates. In stations at trawled areas with shallow excess ^210^Pb profiles, its concentration tended to match the sedimentological discontinuities described above, mirroring sand contents at NF-4 and NF-5 and associated with the upper low-density region at SF-1 and SF-2 (Figs. 2, 3).

At control sites (SF-3, SF-4, SF-5 and SF-6), ^210^Pb exponential-decaying concentration profiles were identified below the surface mixed layers and allowed to calculate apparent maxima sediment accumulation rates of, respectively, 0.74±0.04, 0.51±0.02, 0.26±0.02 and 0.39±0.02 g cm^−2^ y^−1^, corresponding to sedimentation rates of 1.0±0.1, 0.66±0.02, 0.35±0.02 and 0.50±0.02 cm y^−1^ (Table 1). Nonetheless, the remarkable estimated thickness (10–17 cm) of the surface mixed layers of these cores suggests that they could mask the existence of more than one sedimentation rate (see Discussion for details).

### Organic carbon

Organic carbon (OC) content in surface sediments ranged from 0.34% in the topmost cm of core NF-5 to ∼1% at the topmost cm of cores SF-2 and SF-5, disregarding an isolated peak of 1.9% OC observed in SF-1 at 4 cm depth that we attribute to the presence of living fauna in the sample rather than to sedimentary organic matter. Sediment cores from the distal north flank (NF-4 and NF-5) accounted for most of the lowest OC values found in this study.

Regarding the shape of OC vertical profiles, two main patterns can be differentiated. On one hand, decreasing profiles with maximum (0.9–1.0%) OC contents at or near the surface declining progressively with increasing depth. This was the case of the 4 untrawled stations (SF-3 to SF-6), although the trawled NF-1 also seems to fit in the same pattern. On the other hand, at stations NF-2, NF-4, NF-5 and SF-1, minimum OC values occur at or near the sediment–water interface, increasing downcore to a sub-surficial maximum and then tending to relatively stable values. In these cases, the upper layer relatively depleted in OC is approximately coincident with sedimentological contrasts outlined in previous paragraphs, i.e., a low-density upper layer in cores SF-1, NF-1, NF-2 and a coarsened upper sediment layer in cores NF-4 and NF-5. Different patterns can be found in NF-3, which presents a relatively flat OC profile, and in SF-2, which shows a very narrow topmost OC maximum of 1.04% declining very rapidly to stable concentrations below 2 cm depth (Figs. 2, 3).

## Discussion

### Contrasting properties of surface sediments at trawled and untrawled sites

**Figure 4:**
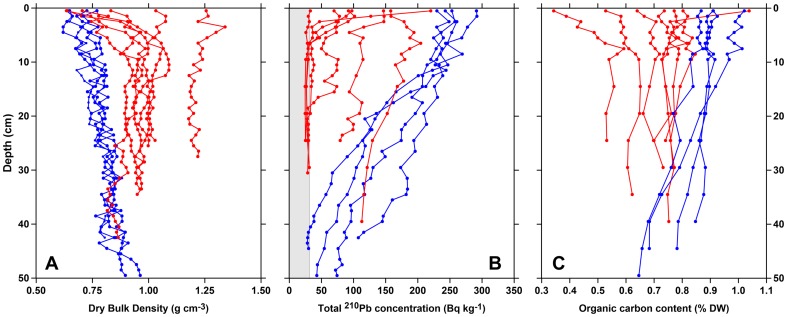
Contrasting sediment properties at trawled and untrawled areas. From left to right, vertical profiles of: dry bulk density (A); total ^210^Pb concentration (B) and organic carbon content (C), in sediment cores collected at the flanks of La Fonera Canyon, grouped according to their position in fishing grounds (red) or control (untrawled) areas (blue). The profile with the highest densities corresponds to core NF-5 from the distal part of the north canyon flank. The shadowed area in the central panel represents supported (in equilibrium with ^226^Ra) ^210^Pb. Concentration values in excess of that threshold indicate net accumulation of recently (within the order of a century) deposited sediments, while ^210^Pb concentrations within the threshold indicate sediments older than 100–150 years. Horizontal error bars in ^210^Pb data are 1-sigma. For the sake of clarity, an isolated value of organic carbon of 1.9% at 4 cm for core SF-1 has been omitted.

The results obtained in this study indicate notable differences between trawled and untrawled sites in terms of the vertical distribution of physical and compositional parameters. To highlight such differences, the vertical profiles of total ^210^Pb concentration, dry bulk density and organic carbon content of sediment cores are grouped and shown in Figure 4, differentiated by color according to their pertinence to trawled or control areas.

It is clear from Figure 4 that surface (0–50 cm) sediments at trawled sites, compared to those from control sites, are denser, depleted in OC and also poorer in ^210^Pb (i.e. ‘older’ in terms of time from deposition).

At the untrawled sites, dry bulk densities increase steadily from surface values ∼0.6 g cm^−3^ to 0.8–1.0 g cm^−3^ towards the bottom of the cores, reflecting the progressive compaction of sediments with depth. This, in combination with the deep horizons of excess ^210^Pb indicates a sedimentary environment where net and relatively steady accumulation of sediments is taking place over modern times. In contrast, at trawled sites, the upper layer of unconsolidated sediment with a dry bulk density <0.7 g cm^−3^ is either very thin (SF-1, SF-2, NF-1, NF-2) or virtually absent (NF-3, NF-4, NF-5). This suggests that erosion has prevailed at these sites, in agreement with the low ^210^Pb inventories and surface concentrations in most of these cores, with the exception of NF-1 and NF-2, which present relatively low surface activities but deep excess ^210^Pb horizons. The low OC content in trawled sediments compared to untrawled ones (Fig. 4) also testifies to the denudation of recent surface sediments on the trawling grounds, which we attribute to the chronic resuspension of sediment induced by the repeated passage of trawling gears.

Together with erosion, ploughing and stirring of sediments can also be invoked to account for the differences seen in Fig. 4, namely the low ^210^Pb and OC concentrations that can result from mixing of recently deposited particles with older, reworked sediments, depleted in unsupported ^210^Pb. Interestingly, lower surface activities and inventories and shallower horizons of excess ^210^Pb are also typical signals of soils altered by agriculture [26]. It is a well-known fact that trawling gears have a daunting capacity to resuspend, disturb and erode bottom sediments. Furthermore, a positive feed-back between trawling disturbance and sediment erosion may occur [27]. Intense sediment resuspension and stirring caused by the repeated passage of trawling gears breaks the natural fabric of sediments and kills or alters the infaunal communities that help to preserve seafloor integrity [1], [20], [28]. This disruption and loss of cohesiveness of the near-surface sediment column results in an increased probability of renewed resuspension, which ultimately denudates the recent sediment cover, in a similar way as agricultural practices and wild fires expose soils to erosion on land [29].

### Trawling-induced textural changes and bed armouring

Trawling disturbance of the seafloor, aside from causing erosion and mixing, may as well induce changes in the grain-size distribution of the remaining sediments, as repeated resuspension favors the sorting of particles according to their settling speeds. In the present study, a coarsening trend toward the surface is observed only in trawled stations SF-1, NF-4 and particularly NF-5 (Fig. 2). Such trends have been observed previously in trawling grounds of shallower areas such as the prodeltas of the Llobregat and Ebro rivers [30], [31], the Gulf of Lions [32] or the Thermaikos Gulf [33].

Therefore, the upward coarsening in cores NF-4, NF-5, SF-1 can be interpreted as the result of sediment winnowing after repeated cycles of resuspension and advection of fine sediments, which leads to progressive bed armouring. Bed armouring is a process that operates on underwater sedimentary beds where fine and coarse grain-sizes co-exist. Under a water flow of sufficient energy or following short-lived sediment resuspension events, the finer fraction of the particle pool is selectively advected by currents and the topmost sediment becomes thus progressively coarser as the fines are lost. As erosion, resuspension and winnowing proceeds, the coarser material is accumulated at the top of the sediment column, gradually forming a shield that prevents further erosion of the underlying finer sediments [34]. Two basic types of bed armouring can be described according to the dominant sorting process [35, and references therein]: in “competence armouring”, fine sediment winnowed by currents that are unable to resuspend coarse particles is the responsible for the progressive coarsening; in “capacity armouring”, more suited to the present case, particles of all size classes present in the mixture can be entrained by the flow, but the finer fraction is selectively swept away further than denser grains, resulting in spatial particle partitioning according to particle settling velocities.

### Organic carbon impoverishment

Concentrations of organic carbon in surface sediments also reflect the impacted or untrawled status of the coring sites. Impoverishment in organic carbon is notable in trawled stations compared to control sites (Fig. 4).

Control sites display a progressive decreasing vertical profile of OC (Fig. 4), as one should expect in marine sediments where steady sedimentation is taking place, i.e., a decreasing gradient of organic carbon concentration from the sediment–water interface to the base of the oxic layer. It is noteworthy how the opposite tendency occurs at most trawled stations: OC varies from relatively low concentrations at the surface to higher contents with depth, usually oscillating around fairly stable values at a given depth. In the case of SF-1 and SF-2, high OC contents are present in a thin topmost unit with very low density, but rapidly decline with depth (Fig. 3).

OC impoverishment of surface sediment by trawling can be the consequence of both the removal of recent, organic-rich sediments, and sediment stirring that increases oxygen penetration and hence the remineralization of buried organic matter. Also, flocculent OC can be winnowed along with the fine sediment fraction and the remaining coarser sediment thus become poorer in OC. It is remarkable how at trawled stations NF-2, NF-4, NF-5 and SF-1, the minimum OC values occurred in the topmost sedimentary unit, suggesting this upper layer is not the result of steady deposition but rather heavily reworked material, as also indicated by low ^210^Pb concentrations and inventories (Figs. 2, 3; Table 1). In the case of NF-3, the absence of ^210^Pb is coherent with a flat OC profile, meaning that all recent sediments have been lost due to enhanced localized erosion (Fig. 2).

These results are coherent with a recent work targeting the same area [19], where it was shown that the nutritional value of sedimentary organic matter of the topmost (5-cm) of sediments along the flanks of La Fonera Canyon is lower at trawled sites compared to untrawled sites, particularly in terms of the amino acid content.

### Sedimentation rates

To contrast the surface ^210^Pb activities found in the sediment cores collected in this study with those expected in the absence of trawling-induced resuspension, we can resort to ^210^Pb concentrations in settling particles collected by sediment traps deployed within La Fonera Canyon at similar depths. Concentrations of ^210^Pb averaged 253±14 Bq kg^−1^ in a near-bottom trap deployed for 8 months at the canyon head (500 m depth) and 266±27 Bq kg^−1^ at another trap at mid-waters (400 m depth in a total water depth of 1200 m) near station NF-3 [11]. We assume these 2 sediment traps were not affected by sediments resuspended by trawling activities, the first because of its location inshore from trawling grounds and the second due to its large distance from the seafloor. These values obtained from sediment traps are also consistent with surface ^210^Pb concentrations found in a study by [25], where topmost ^210^Pb concentrations in NW Mediterranean margins ranged between 185 and 377 Bq kg^−1^ (mean 272±60 Bq kg^−1^) in a depth range 324–861 m. Adopting an average concentration of 259±27 Bq kg^−1^ from the trap data in [11] as the mean activity of newly deposited sediments at depths sampled in the present study, this number fits very well with the surface concentrations at the four untrawled stations, i.e. 200–260 Bq kg^−1^. In contrast to these control sites, trawled stations present significantly lower surface concentrations of ^210^Pb than the adopted (trap-derived) reference, confirming the loss of recent sediments. SF-2, with a topmost ^210^Pb concentration of 191 Bq kg^−1^ may seem an exception to the rule, but it must be noted that such a high activity is restricted to the upper 1 cm, likely associated with a fine ephemeral layer of recently deposited particles overlying reworked and eroded sediments. In fact, excess ^210^Pb concentration declines rapidly to zero at 2–3 cm depth in core SF-2. Even the two trawled stations with relatively deep ^210^Pb horizons and large inventories (NF-1 and NF-2) present surface concentrations that are less than half the “freshly deposited” reference. To account for that low activity, two processes can be invoked, separately or in combination. On one hand, the ^210^Pb content of fresh particles is diluted as intense sediment stirring mixes them with older reworked sediments. On the other hand, in an area of strong particle deposition such as in the vicinity of the canyon head and the proximal north flank [11], [12], canyon trawling-induced sediment erosion may be insufficient to wash away all recently (within roughly a century) deposited sediments (see section 4.5). Therefore, calculation of sedimentation rates was only possible for cores collected from the untrawled area, yielding sediment accumulation rates in the range of 0.3 to 0.7 g cm^−2^ y^−1^, corresponding to sedimentation rates between 0.3 and 1.0 cm y^−1^ (Table 1).

The vertical distribution of excess ^210^Pb in cores NF-1, NF-2, NF-5, SF-1 and SF-2 (Figs. 2, 3) could indicate that these cores are also suited to derive sediment accumulation rates. However, the low ^210^Pb inventories and surface activities, compared to untrawled sites as well as to other cores from similar depths studied by others (e.g., [24], [25]), suggest that this would not be the appropriate interpretation. Lower organic carbon near the surface (in contrast to the situation observed at untrawled sites) also suggests an intense reworking of surface sediments which further precludes the calculation of sediment accumulation rates for these upper layers. It is also noteworthy that in cores NF-4 and NF-5 (and to some extent SF-1), excess ^210^Pb is present only in coincidence with topmost coarsened sediments, declining to virtually zero in the underlying finer and denser sediments. Such an association is contrary to expectations of ^210^Pb enrichment in the finest fraction of sediments (a consequence of increasing ^210^Pb scavenging with increasing specific surface area) as usually encountered in other depositional systems [36, 37 and references therein]. Therefore, we believe that these apparent decay curves are deceptive and are not the consequence of steady sedimentation but of a sorting process that does not necessarily bear any chronological meaning.

Another aspect worthy of discussion concerning sedimentation rates at the control sites is the remarkable thickness of the surface mixed layers, particularly SF-3 and SF-4 (16 and 17 cm respectively). These layers could also be interpreted as indicative of an increase of the sedimentation rates due to trawling activity. Enhanced sediment accumulation rates were found in the canyon axis at 1750 m depth, being related to the down-slope sediment transport generated by trawling-induced sediment gravity flows triggered along the flanks of the canyon [10], [15], [17]. Nonetheless, sediments resuspended at the fishing grounds can also be detached along isopycnals and be advected along the margin and/or along isobaths as intermediate nepheloid layers [38], and in this way, reach the untrawled southern canyon flank enhancing sedimentation rates there.

### Inshore/offshore domains and the relative weight of human/natural disturbances

The results obtained along the flanks of La Fonera Canyon do not only show contrasts between trawled and untrawled areas. Also within the trawled northern flank there are sedimentological and ^210^Pb differences that are likely related to natural processes rather than just to the anthropogenic impact.

The decrease in the sediment remobilization capacity with distance from the shore has been signaled as a control factor for evaluating the relative impact of bottom trawling, as natural processes driving sediment resuspension, transport and accumulation diminish with increasing water depth and distance from nearshore sediment sources [32], [39]. Likewise, sediment inputs are more important in the proximal part of La Fonera Canyon, due to the along-shelf transport of sediments delivered by rivers or resuspended and transported by storms and dense shelf water cascading [11], [12], [13]. Following this approach, [40] suggested a division of the canyon into two hydro-sedimentary domains consisting of an ‘inner’ domain corresponding to the part of the canyon that is incised into the continental shelf and influenced by strong lateral advection of particles, and an offshore ‘outer’ domain where slope and open sea dynamics prevail [11], [40].

The inshore domain along this gradient would be represented by the most inshore stations NF-1 and NF-2, where the losses of superficial sediments by trawling-induced resuspension and erosion are counterbalanced by substantial inputs of fresh sediments, resulting in an intense mixing of old and recently deposited sediments. This is in agreement with the presence of relatively high ^210^Pb inventories, but with ^210^Pb surface activities about half of those measured at control sites. In the case of NF-1, the presence of laminations in its X-radiograph (Fig. 2) suggests that the ^210^Pb profile is not thoroughly mixed and instead represents the truncated profile corresponding to a former accumulation area that is presently being eroded.

As the supply of fresh sediments decline in an offshore direction, the relative importance of net erosion and winnowing increase in NF-4 and NF-5. Core NF-3 represents a turning point in this onshore/offshore gradient, almost lacking any excess ^210^Pb (i.e. virtually all recent, <150 years, deposited sediments are lost) while showing no upwards sediment coarsening. It is worth mentioning that the station NF-3 is located at the head of the Montgrí tributary valley, where frequent trawling-induced sediment gravity flows have been documented downslope, at its confluence with the main canyon axis [10], [17], [38]. In core NF-4, sand content clearly increases in the upper 15 cm of the sediment column with respect to landward stations while the excess ^210^Pb profile indicates thoroughly mixed sediment. The tendency towards increasing coarseness of topmost sediments is further enhanced in station NF-5. The vertical profiles of density, grain-size distribution and excess ^210^Pb of core NF-5 delineate two well-differentiated sedimentary units (a photograph of the fresh sediment tube is available as Figure S2). A coarse surface layer of about 5 cm thickness contrasts sharply with the underlying finer sediment, which consist of overconsolidated mud without any excess ^210^Pb, suggesting that very old (long buried) sediments have almost surfaced due to sustained erosion. The exposure of these deep sediments is not complete due to the bed-armoring effect of the overlying coarser layer (see section 4.2). Regarding the most offshore station NF-6, the extreme stiffness of the bottom sediments prevented the multicorer tubes from penetrating the sediments and/or prematurely fired the multicorer shutter plates. The tubes arrived empty and the legs of the multicorer came aboard covered by a mixture of coarse biogenic debris and extremely stiff mud, suggesting an exacerbation of the situation already observed in NF-5.

**Figure 5:**
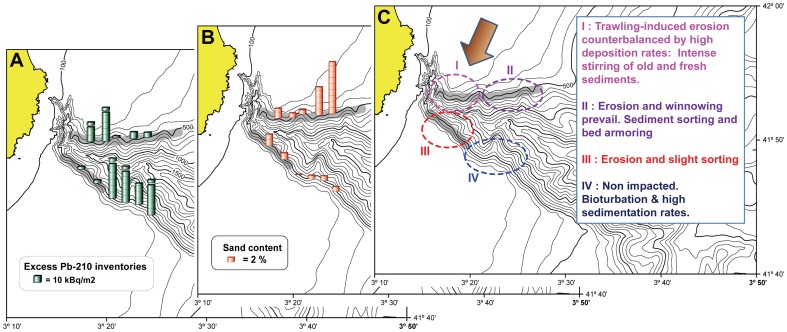
Trawled/untrawled and inshore/offshore gradients in the flanks of La Fonera Canyon. Excess ^210^Pb inventories (A); sand content in the upper 10 cm of the sediment column (B); interpretative summary of the results (C). Cropped bars in the ^210^Pb inventories indicate that the ^210^Pb supported level was not reached and consequently these inventories represent lower estimates of the excess ^210^Pb accumulated at each site. The arrow indicates the approximate position of the main along-shelf sediment transport. Coordinates are East and North degrees/minutes for longitude and latitude respectively.

A conceptual model summarizing the two overlapping gradients (trawled/untrawled and inshore/offshore) of sediment physical properties proposed in this section is presented in Figure 5. Four sub-areas are thus defined: I) an area where trawling-induced resuspension is counterbalanced by high lateral inputs of particles, corresponding to the proximal north flank; II) an area heavily impacted in the distal north flank where intense erosion and/or grain size sorting and bed armoring are taking place; III) the trawled inshore south flank, that presents intermediate properties between the precedent two (erosion is notable and grain size sorting moderate) and finally, IV) a non-impacted area presenting high deposition rates, low dry bulk densities and high surface ^210^Pb. Results from a PCA conducted using the parameters from the upper 10 cm of the sediment column support such a classification (Fig. S3).

### Global implications

The concerns raised by our observations on the flanks of La Fonera Canyon are likely applicable to many continental slope settings of the world, given the wide geographical extension of bottom trawling [6], [9], [10]. Nonetheless, it must be noted that the flanks of this submarine canyon may represent—at least in present times—an extreme case of anthropogenic disturbance of bottom sediments, given the persistence (>60 years) of industrial trawling activities. The peculiar biology of the deep-sea shrimp *Aristeus antennatus* may have contributed to maintaining this target-species fishery near an optimum state of exploitation for decades [41]. Other deep-sea fisheries have collapsed after a few years/decades of intensive exploitation, due to the low recovery rates and high vulnerability to commercial exploitation that often characterizes deep-sea animals and habitats [5], [42]. Therefore, the sustained and chronic (accumulated over several decades) impacts present in this study area can be rather uncommon at a global scale. But, since deep-sea fisheries are continuously expanding, our study could be understood as a foreseeable scenario on incipient deep trawling grounds, should they continue to be trawled persistently for decades. Finally, even if the observed changes in sediment properties may constitute an extreme case, this does not imply that “intermediate” impacts are inconsequential for deep-sea habitats.

## Conclusions

The persistent practice of bottom trawling along the flanks of La Fonera Canyon has altered the sedimentary budget and physical properties of surface sediments. Compared to untrawled sites at similar depths, sediments over fishing grounds show higher degrees of mixing, erosion and sorting and are also impoverished in organic carbon, as a consequence of repeated disturbance by trawling gears. Superimposed on this trend, a seaward tendency towards increasing sediment grain-size and compaction was observed along the northern canyon wall, and attributed to the offshore decreasing availability of fresh sediments which results in enhanced erosion and winnowing prevailing in the distal canyon flank. Our work contributes to the growing understanding and realization of the capacity of deep bottom trawling to stir and erode the seafloor, altering its physical properties and affecting its integrity over large spatial scales, at depths where natural processes can hardly counterbalance these anthropogenic impacts.

## Supporting Information

Figure S1 **Photograph of core NF-1 just after collection.**
(TIF)

Figure S2 **Photograph of core NF-5 just after collection from La Fonera Canyon northern flank at 484 m depth.** The sediment tube presented two distinct layers consisting in a coarse upper layer and very stiff mud below.
(TIF)

Figure S3 **Scatter plot of the two major components of the PCA conducted on four independent normalized variables (sand percentage, dry bulk density, organic carbon content and excess ^210^Pb concentration) from the upper 10 cm of the sediment column.** The first and second components represent 87% of the variance among samples (65% and 22%, respectively). Samples have been identified in the plot using the core number and sampling depth (e.g. NF1-1 means core NF-1 at 0–1 cm). Colors represent the different zones identified in Figure 5, which display a gradient from untrawled sediments (blue samples from zone IV) to more impacted ones (purple and red samples from zone II and III). Surface samples from cores NF-4 and NF-5 deviate from this gradient in an orthogonal direction due to their increased sand content.
(TIF)

Dataset S1 **Sedimentological and radionuclide data for each sediment core used in this study.**
(XLSX)
