# Supplementary figures and images for: Impact of Bottom Trawling on Deep-Sea Sediment Properties along the Flanks of a Submarine Canyon
Source: PLoS One. 2014 Aug 11;9(8):e104536. doi: 10.1371/journal.pone.0104536 (PMC4128664; doi:10.1371/journal.pone.0104536)

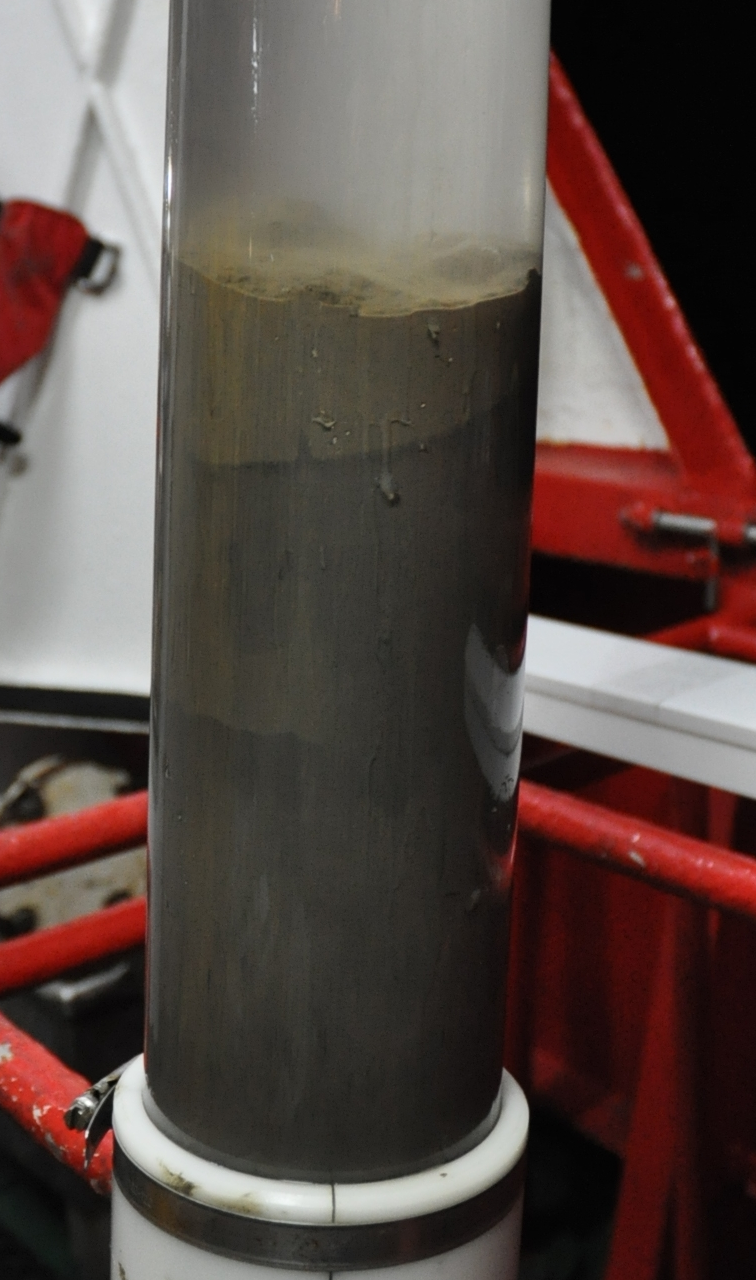

Supplement: Figure S1 — Photograph of core NF-1 just after collection. (TIF) [file pone.0104536.s001.tif]

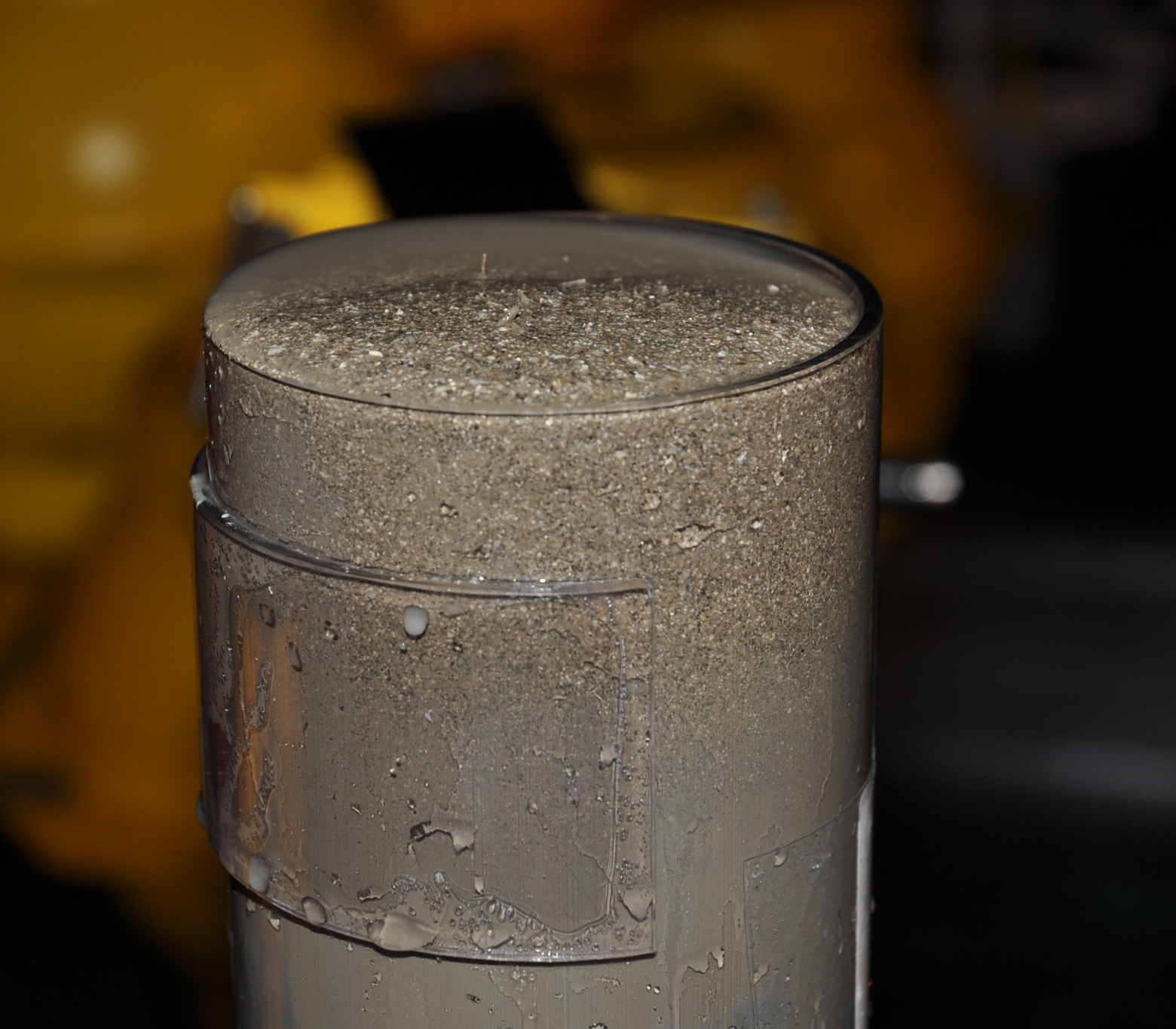

Supplement: Figure S2 — Photograph of core NF-5 just after collection from La Fonera Canyon northern flank at 484 m depth. The sediment tube presented two distinct layers consisting in a coarse upper layer and very stiff mud below. (TIF) [file pone.0104536.s002.tif]

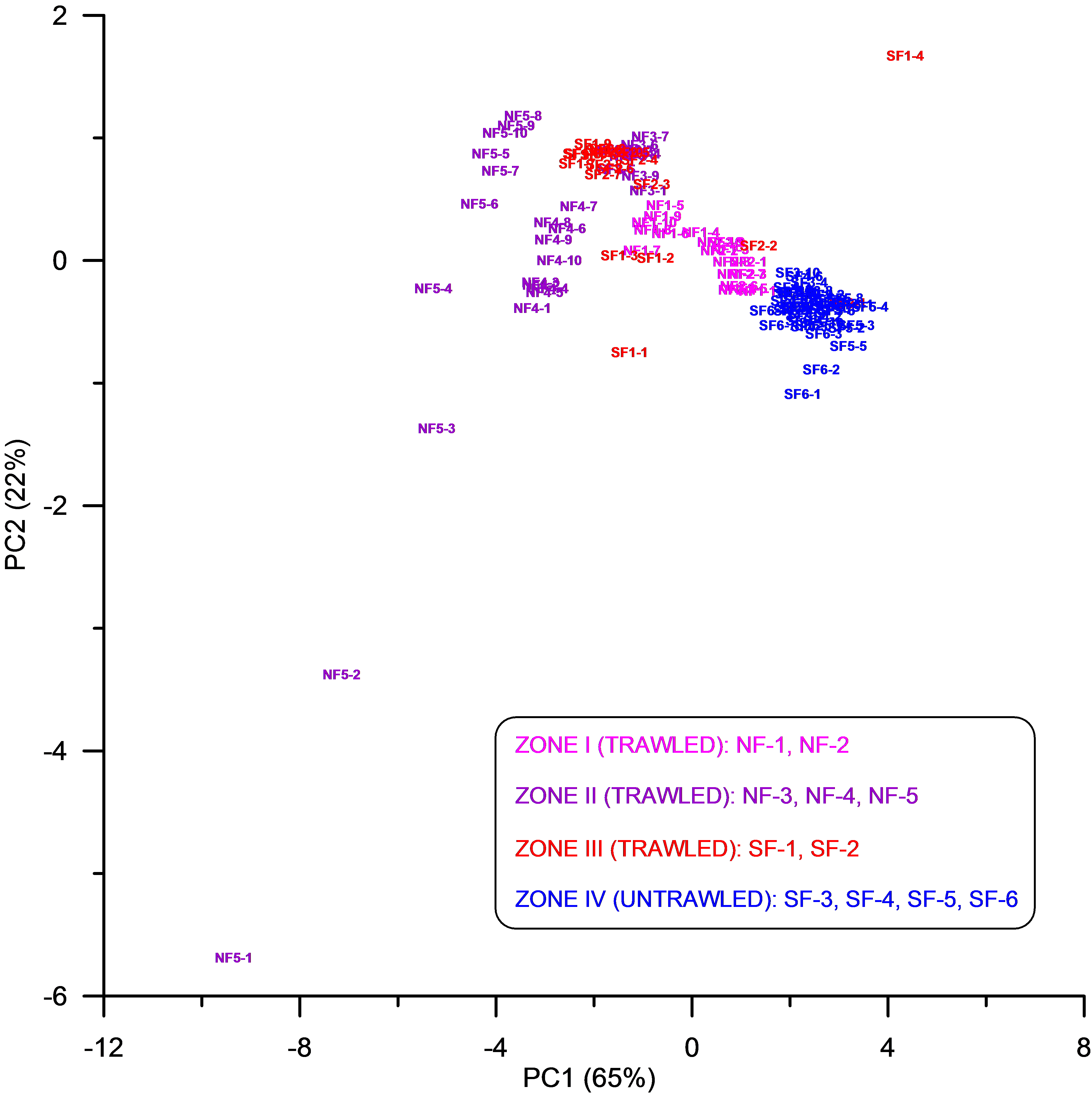

Supplement: Figure S3 — Scatter plot of the two major components of the PCA conducted on four independent normalized variables (sand percentage, dry bulk density, organic carbon content and excess 210Pb concentration) from the upper 10 cm of the sediment column. The first and second components represent 87% of the variance among samples (65% and 22%, respectively). Samples have been identified in the plot using the core number and sampling depth (e.g. NF1-1 means core NF-1 at 0–1 cm). Colors represent the different zones identified in Figure 5, which display a gradient from untrawled sediments (blue samples from zone IV) to more impacted ones (purple and red samples from zone II and III). Surface samples from cores NF-4 and NF-5 deviate from this gradient in an orthogonal direction due to their increased sand content. (TIF) [file pone.0104536.s003.tif]
